# Gender disaggregation of the TB care cascade in Nigeria: a four-year retrospective study 2018–2021

**DOI:** 10.5588/pha.24.0032

**Published:** 2025-06-04

**Authors:** C. Ugwu, C. Aneke, O. Chijioke-Akaniro, J. Kushim, O. Adekeye, G. Kolawole, C. Okoye, J. Bimba

**Affiliations:** ^1^ Department of Clinical Sciences, Liverpool School of Tropical Medicine, UK;; ^2^ Zankli Research Centre, Bingham University, Karu, Nigeria;; ^3^ Faculty of public health and policy, London School of Hygiene and Tropical Medicine, UK;; ^4^ National Tuberculosis, Leprosy and Buruli-Ulcer Control Programme, Department of Public Health, Federal Ministry of Health, Abuja, Nigeria;; ^5^ Department of Community Medicine, Faculty of Clinical Sciences, Bingham University, Karu, Nigeria;; ^6^ Jos University Teaching Hospital, Jos, Nigeria;; ^7^ CARITAS Nigeria.

**Keywords:** gender difference, tuberculosis control, inequality

## Abstract

**BACKGROUND:**

The global TB burden shows significant gender disparity with men and women facing distinct challenges in accessing comprehensive care for TB. A full understanding of the gender dimensions of the TB epidemic is crucial for appropriate policy interventions and we therefore explored gender differences in the TB service cascade in Nigeria.

**METHODS:**

A retrospective gender-based analysis of the TB care cascade was conducted covering the four-year period between 2018–2021. We obtained sex-disaggregated service utilisation data for adults (aged ≥15 years) in 14 states through the monitoring and evaluation systems of the TB control programme. Using a care cascade framework, we present numbers accessing care at each step and gaps for men and women including TB/HIV collaborative services.

**RESULTS:**

Overall, amongst men, 12.3 million visited health facilities, 6 million were screened for TB and 833,483 were identified as presumptive cases, of which 79% were tested for TB. For women, 12.3 million visited facilities, 6.9 million screened and 664,130 identified as presumptive cases, of which 76% were tested. Men exhibited a higher screening gap, whereas women had a higher testing gap, with variations in treatment outcomes across both genders.

**CONCLUSION:**

The TB surveillance system screened more women and diagnosed more men with the disease, with significant missed opportunities and gaps along the continuum of care for both men and women. Targeted policy interventions are required to strengthen surveillance, data systems and to reduce gender inequity across the TB care cascade in Nigeria.

Gender is an important aspect of the global TB epidemic due to its potential role in accelerating the efforts to end this preventable, curable and yet devastating disease. In 2021, WHO data showed that adults accounted for nearly 90% of the new notified cases of TB, with 56.5% being men and 32.5% being women.^1^ With over 200 million people, Nigeria has the largest population in Africa and also the continent’s highest TB burden.^2,3^ The country is one of the 30 high burden TB countries worldwide and shares 87% of the global TB burden with seven other countries.^1^ Nigeria’s nationwide TB prevalence survey showed a national TB prevalence of 524 cases (95% CI: 378–670) per 100,000 population and an estimated incidence rate of 219 cases per 100,000.^4^ A wide gap exists between people who fall sick with TB and those diagnosed and treated as shown by a national Prevalence-Notification ratio of 5.78.^4^ As a result, over the past 5 years, between 60–70% of all those with TB missed out on diagnosis and treatment services.

The sex distribution of TB notification in Nigeria shows a similar pattern to the global picture with adults contributing 93% of notified cases.^1^ In 2020, the estimated incidence of TB was 467,000 TB cases; of whom, 138,591 were diagnosed and notified.^5^ More than half of the notified cases (56%) were men whereas women accounted for a little above one third of cases (37%).^5^ This difference in TB burden can be traced to gender-related factors affecting risk of TB disease and access to care. Although poverty and illiteracy are universal factors associated with TB,^5^ undernutrition, HIV, Diabetes Mellitus, smoking, an aging population and exposure to indoor solid fuel, are established risk factors in Nigeria.^7,8^ Men tend to have more social contacts,^9^ work in high TB-risk settings,^10^ engage more in smoking^11^ and consume more alcohol than women,^12^ factors that contribute to the higher rates of TB and adverse TB outcomes in men. Additionally, men are more likely to migrate for work, leading to more defaults on their TB treatment plans.^13^ Conversely, women may face more TB-related stigma (societal pressures that can lead to divorce or unmarried status).^14^ They also face restrictions in accessing healthcare from sociocultural and economic factors,^15^ which delays diagnosis, and have higher rates of extrapulmonary TB,^16^ which poses screening difficulties and could further reduce diagnosis rates among women.^6^ The impact of high TB risk for men and women are intricately linked as excess burden in men usually implies additional caring roles for the women and increased pressure to earn to support the household.^15^

Nigeria’s National TB Programme (NTP) implements TB-HIV collaboration as part of the TB care cascade. Amongst other components, this ensures that people diagnosed with TB undergo HIV test as part of their TB care. The National HIV adult prevalence of 1.4% shows significant gender variations as women bear the greater burden,^17^ with a prevalence of 1.0% reported in a nationwide survey for men, compared to 1.8% in women.^17^ In 2022, the overall adult TB-HIV co-infection rate was 5.7%.^1^

In recognition of the importance of gender in the TB response, Nigeria developed the Human Rights and Gender Action Plan for Tuberculosis Care and Prevention in Nigeria 2021–2025, to highlight the need to empower women and girls.^18^ Also, the National Strategic Plan for TB Control 2021–2025, recommends further gender analysis of National TB Programme data to fully understand the dimensions of gender disparity in TB and its ramifications.^8^ Although findings from the 2017 National TB Patient Catastrophic Survey and the Nationwide TB prevalence survey^4,19^ suggest a male predominance in TB disease and delay in reaching care, further analysis of the gendered access to TB care is warranted to better inform policy interventions. All these factors point to an urgent need to interrogate the gendered differences in the TB burden in Nigeria. Therefore, we set out to assess the TB cascade over a 4-year period spanning 2018 to 2021 through a gender lens. Given that Nigeria practices TB/HIV collaboration, data on TB/HIV co-infection over the years is also presented.

## METHODS

### Study setting

Nigeria operates a three-tier system of government comprising Federal, State and Local government areas (LGA). There are 774 LGAs, 36 states, and the FCT.^3^ The states are further grouped into 6 Geo-political Zones: North-Central, North-East, North-West, South-East, South-West and South-South.^8^ Similarly, the health system is made up of tertiary, secondary, and primary care levels.^3^ According to the national health facilities registry, there are about 38,678 health facilities in Nigeria; 85.2% of which are primary care centres, and 14.4% and 0.4% are secondary and tertiary level hospitals respectively.^20^ Slightly over a quarter of all registered health facilities are in the private sector.^20^ In 2021, 19,000 health facilities rendered TB services in the country, representing a 47% service coverage. The routinely collected programmatic data used in this study were generated at the local TB Health facilities, which is then transmitted to the LGA TB supervisor, and further transmitted to the State TB Programme. Routinely, data is subjected to quarterly validation at the state level and programmatic review at the zonal level, after which it is forwarded to the national level.

### Study design

**FIGURE 1.**
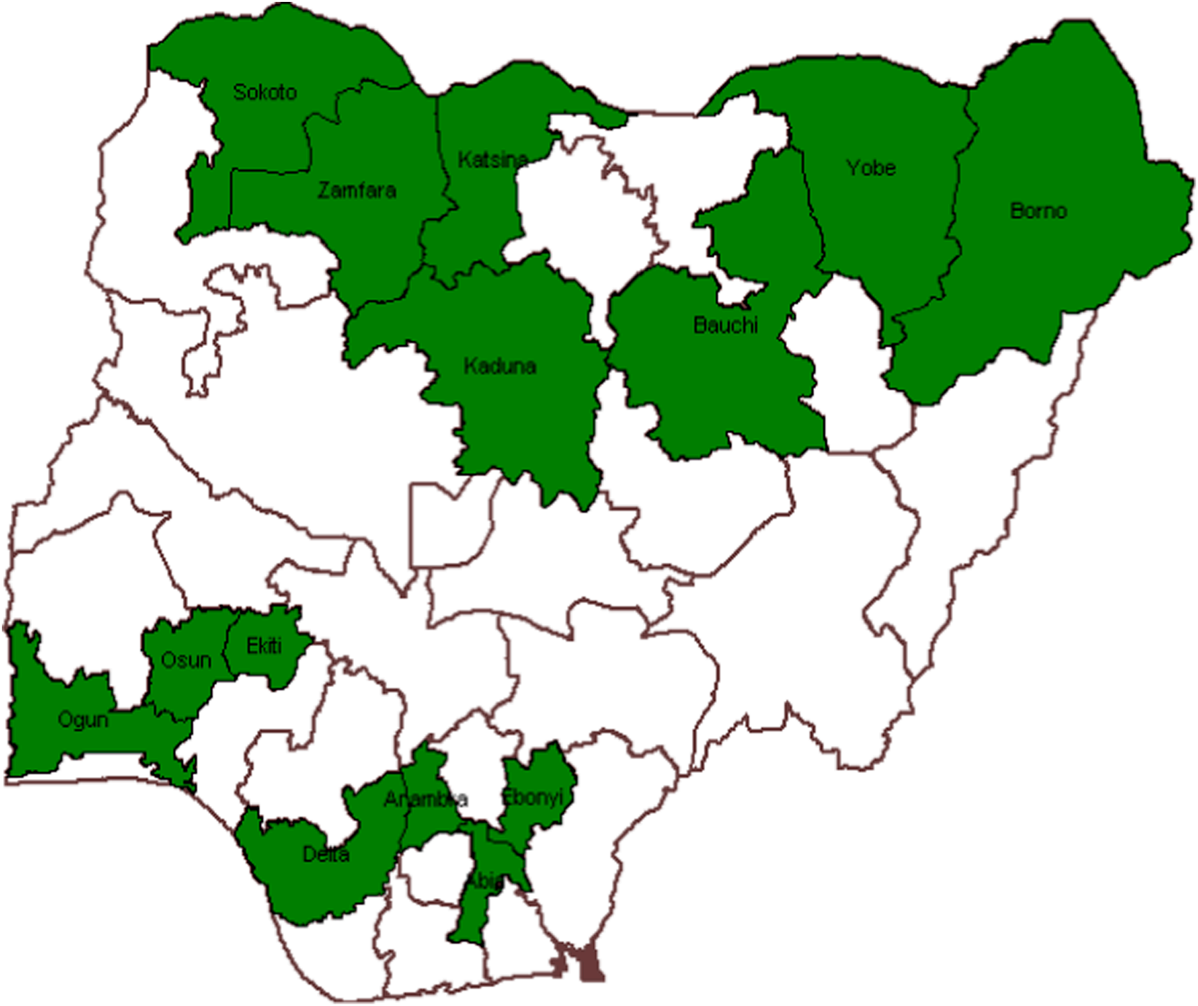
Map of Nigeria showing States (in green) that supplied data.

This study utilized a retrospective cohort analysis study design consisting of NTP monitoring and evaluation (M&E) of TB data from 2018 to 2021 in 14 states (shown in Figure 1) cutting across 5 geopolitical zones in Nigeria. Aggregate data from participants aged 15 years and older was used for this study. This data set is considered to have a nation-wide representative spread for the adult population accessing clinical services at the public and private health facilities in the country across primary, secondary and tertiary care levels.

### Data extraction tool

**FIGURE 2.**
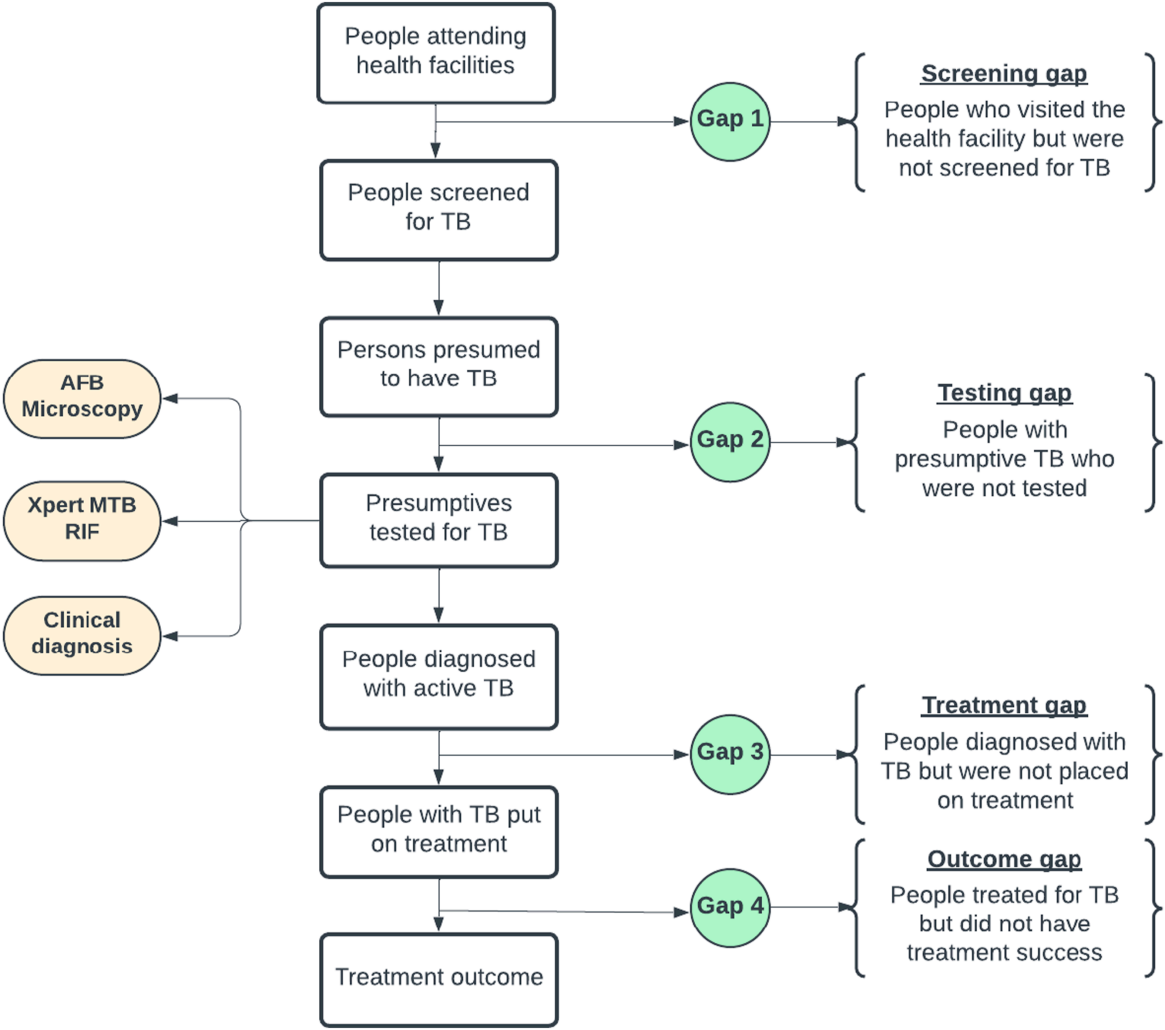
Flowchart of TB care cascade and gaps.

A Microsoft Excel worksheet was developed to collect sex and age-disaggregated data across the TB care cascade from 2018 to 2021. Available data reflects hospital attendance, TB screening, testing, treatment initiation and treatment outcome result. As shown in Figure 2, based on the available data, the research team together with the NTP monitoring and evaluation central team conceptualized key gaps along the care pathway. These include ‘screening gap’ (gap one) to capture people who visited the hospital but were not screened, ‘testing gap’ (gap two) to capture people who were identified as presumptive but were not tested, ‘treatment gap’ (gap three) for those who were diagnosed having TB but not started on treatment, and ‘outcomes gap’ (gap four) for people who were started on treatment but did not achieve treatment success. Treatment success is the sum of those who were cured and those who completed treatment. However, a critical gap that could not be measured exists before the screening gap, and is those who should seek TB services but did not. The key variables include total health facility attendance, TB screening, presumptive identification, TB diagnosis and mode of diagnosis, treatment initiation and treatment outcome among others. Diagnosed and treatment data for 2021 were still being validated during our data collection whilst 2021 cohort outcomes data was not included in the analysis as they were not yet available.

### Data collection and analysis

The data collection tool was distributed to the M&E units of the 36 states and FCT through the NTBLCP M&E unit to facilitate the required data from their existing programme reports from 2018 to 2021. Data for this secondary analysis was collected within 3 months from February to April 2022, with weekly reminders sent by email. Each state monitoring and evaluation officer populated the data collection tool with aggregate data for the state. The number of all persons accessing the various levels of the TB care cascade was described with sex disaggregation for each year. The data were derived from the health facility service registers routinely completed by the healthcare workers. The registers that captured needed information include the out-patient department register, presumptive TB register, laboratory register and the health facility central TB register (treatment register). We have presented the numbers and proportions at each step as well as the gaps in key transition points. The data included in this analysis covers the 14 states of the country that responded to the request.

Given that this research is a secondary analysis of an already existing dataset and that no patient level information was used, written approval to use the data set was obtained from the National TB programme.

## RESULTS

**FIGURE 3.**
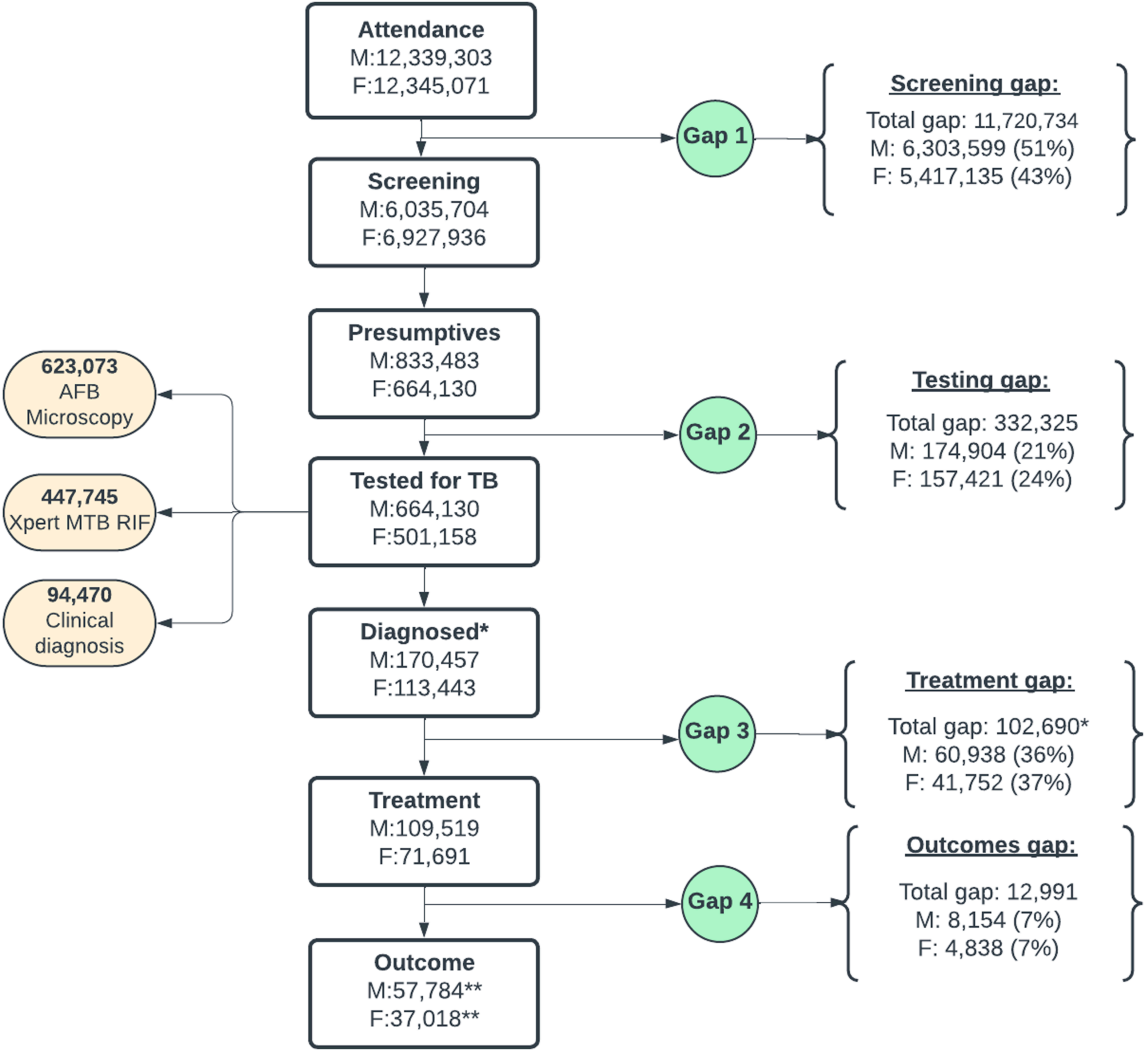
Flowchart of observed TB cascade and gaps in Nigeria 2018–2021. *Diagnosed and Treatment data for 2021 were still being validated during our data collection. **2021 cohort outcomes data was not included in the analysis as they were not yet available.

Over a span of 4 years, healthcare services were provided to 12.3 million men and 12.3 million women at health facilities (Figure 3). Of these, about 6 million males and 6.9 million females were screened for TB, leaving a gap of 51% (6.3 million) for males, and 43% (5.4 million) for females who were not screened for TB respectively. Of all the people screened for TB, 833,483 males and 664,130 females were identified as presumptives, but 664,130, and 501,158 males and females respectively had their samples tested for TB. This left a testing gap of 21% (174,904) for males and 24% (157,421) for females. Figure 3 also shows that 170,457 males and 113,443 females were diagnosed with TB, out of which 36% (60,938) of males and 37% (41,752) of females were not put on treatment. Among those placed on treatment, 94,803 achieved positive treatment outcome, whereas 12,991 had a negative treatment outcome. 57,784 males had positive treatment outcome with 8,154 (7% of males put on treatment) having negative treatment outcomes. For females, 37,018 achieved positive treatment outcomes whereas 4,838 (7% of females put on treatment) had negative treatment outcomes. The treatment outcome numbers exclude the 2021 cohort.

**FIGURE 4.**
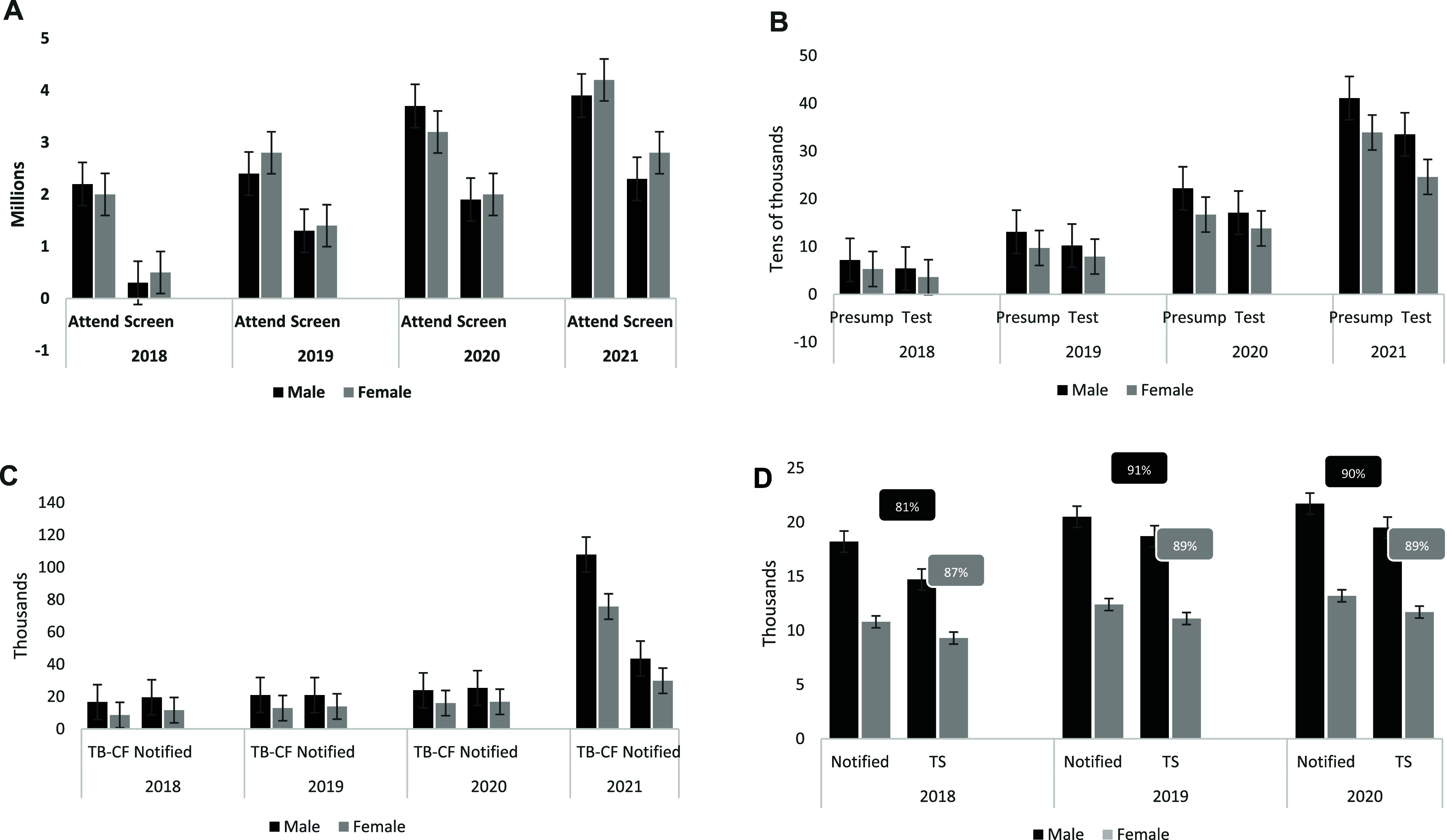
Gender disaggregated trend of access to stages of TB care cascade 2018–2021. A: Trend of gender disaggregated attendance and screening. B: Trend of gender disaggregated presumptive and tested. C: Trend of gender disaggregated TB-CF and notified. D: Trend of gender disaggregated notified, TS and TSR. TB-CF = TB Case finding: Referring to number diagnosed with TB. In Nigeria this number differs from ‘Notified’ as the country considers Notification as people put on treatment and not merely being diagnosed with TB. TS = Treatment success: Referring to people classified as either ‘Cured’ or ‘Treatment completed’ at the end of treatment. Calculated by the sum of people with ‘Cured’ and ‘Treatment completed’ outcomes. TSR = Treatment Success Rate: Referring to the proportion for all the people put on treatment that achieved either ‘Cured’ or ‘Treatment completed’ at the end of treatment. In this figure TSR is shown as the percentages in the rounded rectangles above the treatment success bars.

The 4-year gender-disaggregated data for attendance at health facilities and TB screening is show in Figure 4A. The attendance for each gender was about 2 million in 2018, with men marginally above women, while screening was higher in women in the same year. In 2019 and 2020, there was an increase in the number of attendances to health facilities as well as screening for men and women, with more women than men in both parameters. For 2021, it depicts the highest numbers in both attendance to facilities and screening for both genders, with women having about 4 million for attendance and almost 3 million for screening compared with men having around 3.7 million and 2.5 million for attendance and screening, respectively. Overall, there was an increase in the number of people of both genders who attended and were screened over the 4-year period; however, more women were screened than men. The trend for presumptive and tested people disaggregated by gender over the 4-year period shows a steady rise in both parameters for both genders but associated with male predominance (see Figure 4B). In 2021, both presumptives and tested doubled (from about 389,913 to almost 751,545 for presumptives and from 310,203 to about 581,871 for tested) between 2020 and 2021. TB case finding and case notification disaggregated by gender over the 4-year period, depicts a clear male predominance throughout (Figure 4C). In 2021, there was a marked increase in the trend of case finding (183,658 vs. 33,414) and notification (73,416 vs. 35,931) compared to the preceding three-year average. The trend over a 3-year period for TB case finding and treatment success disaggregated for gender is shown in Figure 4D. The parameters under review saw a gradual increase with each successive year, with men leading in notification over the period. The treatment success rates (TSR) were between 80–90% for both genders over the three-year period, but there was a noticeable increase in the TSR between 2018 and the succeeding two years.

The gender-disaggregated TB/HIV co-infection rates between 2018–2021 are shown in the Table. A total of 283,900 persons were diagnosed with TB, with men accounting for 60% while 40% were women, with an overall co-infection rate of 3.4%. In 2018, 15% of women who fell ill with TB were HIV-positive compared to 7.3% of men, even though more men were diagnosed with TB in that year. Subsequently, the co-infection rate in women fell slightly to 12.7% in 2019, while that of men marginally increased to 7.8%. The following 2 years saw substantial reductions in TB/HIV co-infection rates, with 5.2% and 1.2% in women, and 4.2% and 0.9% in men, respectively.

**TABLE.** Four-year TB-HIV co-infection rate in Nigeria.

		Male	Female	Total
2018	TB Cases Diagnosis	17,610	8,613	26,223
	HIV positive	1,288	1,296	2,584
	Co-infection rate	7.3%	15%	9.9%
2019	TB Cases Diagnosis	21,043	12,965	34,008
	HIV positive	1,646	1650	3296
	Co-infection rate	7.8%	12.7%	9.7%
2020	TB Cases Diagnosis	23,920	16,091	40,011
	HIV positive	1,002	838	1,840
	Co-infection rate	4.2%	5.2%	4.6%
2021	TB Cases Diagnosis	107,884	75,774	183,658
	HIV positive	922	877	1,799
	Co-infection rate	0.9%	1.2%	1%
Four-year Total	TB Cases Diagnosis	170,457	113,443	283,900
	HIV positive	4,858	4,661	9,519
	Co-infection rate	2.8%	4.1%	3.4%

## DISCUSSION

Overall, our gender-based care cascade analysis of TB data in Nigeria from 2018 to 2021 suggests health facility and outreach attendance and screening has improved over the years. Whereas men predominate testing, and treatment numbers, there are substantial gaps in key transition points of the TB care cascade. In addition, HIV associated TB, which affected more women, showed a steady decline over the period. In comparison to men, more women were screened for TB within the 4-year period. This is unsurprising as women tend to make more contact with the health system over their life course, as demonstrated in various studies.^21,22^ Also, men are known to experience limited access to healthcare,^23^ and may delay hospital visits due to several reasons including casualization of illness,^13^ or prioritizing work over treatment.^24^ Notably, almost half of the attendees missed out on screening, with men having a 51% gap whereas 43% of women were not screened. This gap represents a significant missed opportunity to identify presumptives among sick persons at health facilities. Factors related to healthcare worker capacity, facility clinic flow, availability of screening at each service delivery points, and poor documentation practices may have contributed to this gap and thus lowering the sensitivity of passive surveillance.

Our study showed a progressive increase in the number of persons with presumptive TB and those tested for both men and women with a remarkable rise in 2021. Men accounted for the majority in both parameters. A national TB intervention that screened over 21 million people reported that men constituted about half of the 1.9 million presumptives identified across community and facility-based interventions.^25^ However, it showed that men account for more presumptives in targeted interventions with x-ray enhanced screening. This is in keeping with findings from the Nigerian national TB prevalence survey^4^ and the WHO global TB reports^1,5^ as well as other studies within the country.^26-28^ Over three hundred thousand people identified with symptoms were not tested (testing gap) and the greater percentage of them were men. A mixed-methods study done in Nigeria to estimate the gaps along the DR-TB care cascade identified sub-optimal coverage of TB services, shortfall in skilled laboratory staff, and missed diagnosis due to low index of suspicion as key barriers leading to this testing gap.^29^ The marked increase in the numbers seen in 2021 in both genders can be explained by the post-COVID-19 rebound and the strategic programmatic prioritization of active case-finding approach by the NTP.^30^ That policy was rigorously pursued along with a national policy on statutory screening of all OPD patients for TB symptoms.^31^ The result yielded larger number of persons with symptoms who might not have visited TB clinics. These improvements need to be strengthened to maintain and consolidate these gains.

The Nigeria TB programme prioritizes TB notification as a key performance indicator for tracking TB control rather than people diagnosed. This is because the number of people diagnosed with the disease usually includes double- and triple-counting of same individuals as they may visit different testing labs to confirm their diagnosis. As a result, it is quite likely that the data for gap three is an overestimate. Additionally, it suggests a positivity rate of 24% amongst presumptives which is more than double what is common for our setting.^7,21,25^ Per our findings, men dominated TB case finding and notified cases across all the years reviewed with notable increase in 2021. Despite more women attending health facilities and leading the numbers among the screened, more men were diagnosed and notified throughout the assessed period. This agrees with available data from the prevalence survey that showed that men have twice the burden of TB when compared to women.^4^ Some well documented factors linked with their greater involvement in smoking and drinking habits and high risk occupations, social contact patterns, health seeking behavior amongst others may contribute to this.^9-12^ The STOP TB Gender Assessment of Nigeria report and other studies highlight the importance of active systematic screening approaches to detect more men with TB.^6,23,32^

Over the three years for which cohort outcomes data were available, men and women had similar treatment outcomes with men having slightly higher negative outcomes. Although the proportions are similar, the raw numbers plotted in figure 4D shows that more men^4,8,15^ had negative outcomes than females (4,838). One of the key contributors of negative outcomes ‘lost to follow-up’ has be found to be more common amongst men.^33,34^ Challenges related to socioeconomic and socio-cultural factors have been cited as responsible for the difficulty men face in adhering to care.^6^ Enhanced adherence counselling and clinical decision making inclusive of men’s opinions will be important in improving their sustainment on care. Additionally, women and men will also benefit from socio-economic support to ease challenges associated with treatment adherence.^6^ In our study women bore a higher burden of HIV associated TB as has been previously reported in our setting.^35,36^ This higher co-infection rate in women is intricately linked with the social position of women and prevalent gender power dynamics in our patriarchal society.^15^ The overall decline in the national co-infection rate over the time period is reflective of findings from the recent National HIV prevalence survey.^37^

A limitation is that, although we reached out to all the 36 states and the FCT, we received usable data from only the 14 states included in this analysis. A key challenge was the lack of sex disaggregated data in some key indicators at the state and national levels implying that participating states had to reach back to the LGA and health facility levels to be able to complete the study data collection tool. This may have introduced errors within the data. Non-usage of electronic medical records implied that individual level data could not be used for a more precise analysis. Additionally, we were limited by our inability to measure an important gap (gap zero) that should show the persons who should seek TB services but did not. Our cascade also assumes that the WHO four-symptom screening used at health facilities is able to detect all presumptives, and that people who tested for TB all received their results. Both scenarios are unlikely the case. Furthermore, covid-19 associated disruptions may have affected the findings of this study. We acknowledge these weaknesses and have thus been careful in the conclusions reached from our findings. Finally, our analysis did not include TB preventive therapy due to data availability issues. Nevertheless, to our knowledge, this is the first major gender-based cascade analysis at this scale in Nigeria and provides key recommendations for improvement of access for men and women.

## CONCLUSION

Our work highlights gender inequity in the TB care cascade in Nigeria with implications for programme design and how policy interventions could be targeted. Specifically, approaches to enhance TB screening amongst men will be beneficial. Given the role of gender and other social determinants in the TB epidemic, more research and better data are necessary to fully understand the dimensions of its interactions. Although granular data is collected at the point of service delivery, some important details are not included in the collated versions transmitted upwards to state and national levels. Existing data collation tools can be improved to include sex disaggregation at all levels of the cascade from hospital attendance to treatment outcomes. Electronic data capturing and transmission systems with robust data warehousing need to be optimized and strengthened. Additionally, sustainable social protection systems to support individuals affected by TB in improving access to services and sustainment on care are crucial.

## References

[1] World Health Organisation. Global Tuberculosis Report. Geneva, Switzerland: WHO, 2022.

[2] United Nations, Sustainable Development Goals.

[3] The National Strategic Plan for Tuberculosis Control. Nigeria, 2015.

[4] MOH NTBLCP. First National TB Prevalence Survey Report. Nigeria: Ministry of Health, Nigeria; 2012 p. 100.

[5] World Health Organisation. Global Tuberculosis Report. Geneva, Switzerland: WHO, 2021.

[6] Stop TB Partnership; UNOPS. TB GENDER ASSESSMENT IN NIGERIA REPORT. Nigeria; 2018 p. 74.

[7] MOH, NTBLCP. National Epidemiological Analysis 2020. Abuja, Nigeria; 2020.

[8] MOH NTBLCP. The National Strategic Plan for Tuberculosis 2021-2025. Nigeria: Ministry of Health, Nigeria; 2020 p. 278.

[9] Dodd PJ, Age- and Sex-Specific Social Contact Patterns and Incidence of Mycobacterium tuberculosis Infection. Am J Epidemiol. 2015 Dec 8;kwv160. 10.1093/aje/kwv160 PMC4706676 26646292

[10] Marçôa R, Tuberculosis and gender – Factors influencing the risk of tuberculosis among men and women by age group. Pulmonology. 2018;24(3):199-202. 29754721 10.1016/j.pulmoe.2018.03.004

[11] Aigbiremolen, AO Cigarette Smoking and Adolescent Health: A Survey of Selected Senior Secondary Schools in Ekpoma. IJCR. 2013;2(2):28-33.

[12] World Health Organisation. Fact Sheets: Alcohol. Geneva, Switzerland: WHO, 2024.

[13] Chikovore J, ‘For a mere cough, men must just chew Conjex , gain strength, and continue working’: the provider construction and tuberculosis care-seeking implications in Blantyre, Malawi. Global Health Action. 2015 Dec;8(1):26292. 25833138 10.3402/gha.v8.26292 PMC4382597

[14] Kumari Indira KS, Mathew N. TB related stigma and gender disparity among unaffected population in central Kerala, a survey. Indian J Tuberc 2023;70(2):168-75. 37100573 10.1016/j.ijtb.2022.03.028

[15] Onifade DA, Gender-related factors influencing tuberculosis control in shantytowns: a qualitative study. BMC Public Health. 2010;10(1):381. 20587044 10.1186/1471-2458-10-381 PMC2910677

[16] Oshi SN, Investigating gender disparities in the profile and treatment outcomes of tuberculosis in Ebonyi state, Nigeria. Epidemiol Infect. 2015;143(5):932-42. 25355040 10.1017/S095026881400291X PMC9507156

[17] Patel HK, Performance of HIV rapid testing algorithm in Nigeria: Findings from a household-based Nigeria HIV/AIDS Indicator and Impact Survey (NAIIS). PloS Glob Pub Health 2022;2(7):e0000466. 36962526 10.1371/journal.pgph.0000466 PMC10021238

[18] DPH, NTBLCP. Human Rights and Gender Action Plan for Tuberculosis Care and Prevention in Nigeria 2021–2025. Nigeria: Ministry of Health, Nigeria; 2020 p. 39.

[19] MOH N. National TB Catastrophic Survey 2017. Abuja, Nigeria; 2017. Report No. 1.

[20] Federal Ministry of Health, Department of health Planning Research and Statistics. List of Registries.

[21] Yeatman S, Chamberlin S, Dovel K. Women’s (health) work: A population-based, cross-sectional study of gender differences in time spent seeking health care in Malawi. PLoS ONE. 2018 Dec 21;13(12):e0209586. 30576388 10.1371/journal.pone.0209586 PMC6303093

[22] Ogbudebe C, Identifying Hot Spots of Tuberculosis in Nigeria Using an Early Warning Outbreak Recognition System: Retrospective Analysis of Implications for Active Case Finding Interventions. JMIR Public Health Surveill. 2023 Feb 8;9:e40311. 36753328 10.2196/40311 PMC9947752

[23] Horton KC, Sex Differences in Tuberculosis Burden and Notifications in Low- and Middle-Income Countries: A Systematic Review and Meta-analysis. PLoS Med. 2016;13(9):e1002119. 27598345 10.1371/journal.pmed.1002119 PMC5012571

[24] Daniels J, Masculinity, resources, and retention in care: South African men’s behaviors and experiences while engaged in TB care and treatment. Social Science & Medicine. 2021 Feb;270:113639. 33493956 10.1016/j.socscimed.2020.113639 PMC8445063

[25] Babayi AP, Improving TB control: efficiencies of case-finding interventions in Nigeria. Public Health Action. 2023;13(3):90-6. 37736578 10.5588/pha.23.0028 PMC10446662

[26] Adejumo OA, Prevalence of rifampicin resistant tuberculosis and associated factors among presumptive tuberculosis patients in a secondary referral hospital in Lagos Nigeria. Afr H Sci. 2018 Aug 16;18(3):472. 10.4314/ahs.v18i3.2 PMC6307017 30602977

[27] Ibrahim MM, Trends in the incidence of Rifampicin resistant Mycobacterium tuberculosis infection in northeastern Nigeria. Scientific African. 2022 Sep;17:e01341.

[28] Danlami MB, Aliyu B, Samuel G. Incidence of rifampicin-resistance presumptive tuberculosis cases among outpatients in Kebbi state, Nigeria. AJID. 2021 Jan 15;15(1):47-52. 33884358 10.21010/ajid.v15i1.6 PMC8047284

[29] Oga-Omenka C, Understanding the gaps in DR-TB care cascade in Nigeria: A sequential mixed-method study. J Clin Tuberc Other Mycobact Dis. 2020;21:100193. 33102811 10.1016/j.jctube.2020.100193 PMC7578750

[30] FMOH, USAID. Nigeria Federal Ministry of Health and USAID Statement of Partnership. Nigeria: FMOH, USAID; 2019 Aug p. 17.

[31] David. The Sun Nigeria. 2018 [cited 2023 Sep 13]. Adewole recommends TB screening before employment. Available from: https://sunnewsonline.com/adewole-recommends-tb-screening-employment/

[32] Squire SB, ’Lost’ smear-positive pulmonary tuberculosis cases: where are they and Fwhy did we lose them? Int J. Tuberc Lung Dis 2005;9:25-31. 15675546

[33] Htwe KK, Pre-treatment loss to follow-up and treatment delay among bacteriologically-confirmed tuberculosis patients diagnosed in Mandalay Region, Myanmar. Trop Med Health. 2019 Dec;47(1):30. 31073273 10.1186/s41182-019-0154-9 PMC6498628

[34] MacPherson P, Pre-treatment loss to follow-up in tuberculosis patients in low- and lower-middle-income countries and high-burden countries: a systematic review and meta-analysis. Bull World Health Organ. 2014;92(2):126-38. 24623906 10.2471/BLT.13.124800 PMC3949536

[35] Affusim CC, Kesieme E, Abah VO. The Pattern of Presentation and Prevalence of Tuberculosis in HIV-Seropositive Patients Seen at Benin City, Nigeria. ISRN Pulmonology. 2012 Mar 12;2012:1-6.

[36] Sariem CN, Tuberculosis treatment outcomes: a fifteen-year retrospective study in Jos-North and Mangu, Plateau State, North - Central Nigeria. BMC Public Health. 2020;20(1):1224. 32781994 10.1186/s12889-020-09289-x PMC7422002

[37] Federal Ministry of Health, Nigeria. Nigeria HIV/AIDS Indicator and Impact Survey (NAIIS) 2018: Technical Report. Abuja, Nigeria. October 2019.

